# Associations among Mammary Ultrasound Measurements, Milk Yield of Non-Dairy Ewe Lambs and the Growth of Their Single Lambs

**DOI:** 10.3390/ani11072052

**Published:** 2021-07-09

**Authors:** Emmanuelle Haslin, Rene A. Corner-Thomas, Paul R. Kenyon, Sam W. Peterson, Stephen T. Morris, Hugh T. Blair

**Affiliations:** School of Agriculture and Environment, Massey University, Palmerston North 4474, New Zealand; r.corner@massey.ac.nz (R.A.C.-T.); p.r.kenyon@massey.ac.nz (P.R.K.); s.peterson@massey.ac.nz (S.W.P.); s.t.morris@massey.ac.nz (S.T.M.); h.blair@massey.ac.nz (H.T.B.)

**Keywords:** gland cistern, ultrasonography, parenchyma, fat pad, udder morphology

## Abstract

**Simple Summary:**

Mammary internal structures were associated with milk yield in mature dairy ewes and lamb growth in ewe lambs. This experiment was designed to examine the association between mammary ultrasound measurements and milk yield in ewe lambs and the accuracy of using mammary ultrasound measurements to predict single lamb growth rates to weaning. Mammary internal structures were measured in 45 single-bearing ewe lambs at day 110 of pregnancy, week three (W3), five (W5), and seven (W7) of lactation and at weaning (L69). The ewe lambs were milked once at W3, W5 and W7 and the single lambs were weighed at birth, W3, W5, W7, and L69. The predictions of milk yield were moderate, and the predictions of lamb growth were high to moderate, indicating that mammary ultrasound was more accurate in predicting lamb growth than milk yield. Further investigations are required to identify better indicators of milk yield in ewe lambs.

**Abstract:**

Mammary cistern size was positively correlated with milk yield of mature dairy ewes, but the association in ewe lambs is unknown. This experiment aimed to examine the associations between mammary ultrasound measurements and the milk yield of ewe lambs at one year of age and to determine the accuracy of using maternal mammary ultrasound to predict single lamb growth rates. Single-bearing ewe lambs (n = 45) were randomly selected and 30 were milked once at weeks three (W3), five (W5), and seven (W7) of lactation. Mammary ultrasound scans were performed at day 110 of pregnancy, W3, W5, W7, and weaning (L69). Single lambs (n = 30) were weighed at birth and at each mammary scanning event. Udder measurements explained 26.8%, 21.4%, and 38.4% of the variation in milk yield at W3, W5 and W7, respectively, and 63.5% and 36.4% of the variation in single lamb growth to W3 and to L69. This ultrasound technique was more accurate in predicting single lamb growth to W3 than milk yield and may enable the identification of pregnant ewe lambs whose progeny would have greater growth rates. More research is needed to identify accurate indicators of superior milk yield and determine whether ultrasound could be used to select ewe lambs.

## 1. Introduction

Ultrasound scanning is a widely utilized, non-invasive method to examine the mammary glands of ewes and their internal structures [1,2,3,4,5]. Ultrasonography can also be used for the examination and diagnosis of sheep mammary diseases [2,3,6,7] and as a technique for animal selection based on mammary structures [8,9,10]. Currently, most studies have investigated the mammary glands of dairy breed ewes, focusing on the relationship between mammary gland cistern (*Sinus lactiferous*) size and milk production [4,7,8,11], and the impacts of management practices such as milking intervals [9] or the drying-off procedure [1] on the mammary gland. A small number of studies have used ultrasonography to examine the mammary glands of dual-purpose meat and wool breeds [10,12,13].

Mammary gland cistern size measured using ultrasound has been positively correlated with milk production in mature dairy ewes [4,5,7,8,11] and in non-dairy mature ewes [13]. In addition, Barbagianni et al. [2] reported a negative association between parenchymal greyscale values on day 145 of pregnancy and the quantities of milk collected after lambing in three- to five-year-old dairy ewes. The authors also reported a negative association between the greyscale intensity values of the parenchyma three and five days after lambing on ultrasound images and milk quantities collected on the same day. Collectively, these associations suggest it would be possible to use udder ultrasound measurements to select mature ewes with the greatest potential for milk production. In dairy heifers, the proportion of secretory tissue in the parenchyma measured by ultrasound 15 to seven days prior to calving was highly correlated with milk production over 100 days of lactation (r = 0.80) [14]. However, the association between ultrasound measurements of the mammary gland structures and the milk production of dairy and non-dairy ewe lambs during their first lactation is unknown.

Torres-Hernandez and Hohenboken [15] reported that milk production was positively associated with lamb growth, particularly in early lactation. van der Linden et al. [16] noted that single lamb growth rates during the first two weeks of lactation were poorly predicted, and were only moderately predicted by milk yield in week four of lactation. It is possible that ultrasound measurements of the mammary gland structure could be used as an indirect indicator of lamb growth to weaning. Haslin et al. [10] reported the impacts of a heavier live weight of non-dairy ewe lambs at breeding on their mammary gland development during pregnancy and lactation, and the association between mammary gland structures and progeny growth to weaning. The depth of the mammary gland cistern at day 29 of lactation was moderately positively correlated with single lamb growth from birth to 29 days of age and from birth to weaning (100 days of age). In addition, the depth of the mammary parenchyma at 107 days of pregnancy was positively associated with single growth rates to weaning. These mammary measurements could be a potential means of selecting non-dairy ewe lambs likely to wean heavier lambs.

The first objective of this experiment was to examine the associations between ultrasound measurements of the mammary gland of non-dairy ewe lambs rearing single lambs and their milk production. It was hypothesised that, as reported in mature dairy ewes, the depth of the gland cistern would be positively correlated with milk yield of non-dairy ewe lambs. The second objective was to determine if the ultrasound measurements of the mammary gland was an accurate and non-invasive method to assess the milk yield of non-dairy ewe lambs and the growth of their single lambs to weaning at approximately 69 days of age.

## 2. Materials and Methods

All animal handling procedures were approved by the Massey University Animal Ethics Committee (MUAEC 19/49). The experiment was conducted at Massey University Riverside Farm (latitude: 40°50′35″ S, longitude: 175°37′55″ E), 10 km north of Masterton, and Massey University Keeble Farm (latitude: 40°24′03″ S, longitude: 175°35′51″ E), 5 km south of Palmerston North, New Zealand.

### 2.1. Experimental Design

Romney ewe lambs were bred at seven months of age for two periods of 17 days (P0 to P34). At pregnancy diagnosis (P94; 08/08/2019), 45 ewe lambs were randomly selected from ewe lambs successfully mated in the first 17 days of the breeding period and identified as carrying a single lamb. Only single-bearing ewe lambs were selected as single births occur more frequently than twin-bearing in ewe lambs [17]. The fleece of the ewe lambs was removed at P102 (16/08/2019) and ewes were transported to Keeble Farm at P105 (19/08/2019) for the remainder of the experiment.

From the start of breeding (P0) to the weaning of their progeny (L69), ewe lambs were grazed using a rotational grazing system on ryegrass (*Lolium perenne* L.) and white clover (*Trifolium repens* L.) pasture under New Zealand grazing conditions. Ewe intake was considered to be unrestricted as pre-grazing pasture covers were maintained above 1200 kg DM/ha [18]. After moving ewes to Keeble farm, the mean pre-grazing mass offered during pregnancy was 1885 ± 100 kg DM/ha. During lactation, the pre-grazing covers of pastures were recorded every two-weeks from P143 (26/09/2019) to weaning and was on average 2347 ± 159 kg DM/ha.

At P143 (26/09/2019), all ewe lambs were moved to their lambing paddock (n = 45; approximately 18.9 ewe lambs/ha). In order to milk all ewe lambs at the same stage of lactation, ewe lambs were divided into three different milking groups based on day of parturition (Table 1). Ewe lambs were milked once per week during each of week three (13 to 20 days of lactation; W3), five (27 to 34 days of lactation; W5) and seven (41 to 48 days of lactation; W7) of lactation (Table 1). Ewe lambs that did not lamb (n = 2) or whose lamb died at birth or during lactation (n = 12) were excluded from the experiment. Lambs were weaned at approximately 69 days of age (L69; 19/12/2019).

### 2.2. Animal Measurements

#### 2.2.1. Udder Scores

Ewe lamb udder scores and morphological measurements were performed at P110 and L69 after ultrasound scanning using the method described by Haslin et al. [10]. Briefly, the scoring system included the palpation of both udder halves and teats and assessed udder symmetry and depth. Ewe udder and teat palpations were performed in a sitting position to allow access to the udder and udder symmetry and udder depth were assessed in a standing position [19].

Morphological measurements were taken while ewe lambs were standing. Morphological traits were measured as described by Haslin et al. [10] and included udder circumference (UC, cm) and the height of each udder half (cm). Udder volume (UV, cm^3^) was calculated using UC and an average of udder height (UH, cm) according to the method of Ayadi et al. [20]:

R = UC/2π

UV = π × R^2^ × UH

where UV = udder volume (cm^3^); π = 3.14159; R = radius (cm); UH = udder height (cm); UC = udder circumference (cm).

#### 2.2.2. Ultrasound Scanning

Udder ultrasound scans were performed by a single operator at 110 days of pregnancy (P110), at week three (W3), five (W5) and seven (W7) of lactation and at weaning (approximately 69 days of lactation; L69). The ultrasound method was described in detail by Haslin et al. [10]. Ultrasound scans were not performed on ewe lambs that did not lamb (n = 2), who had died (n = 1) or whose lambs had died (n = 12). In lactation, ultrasound scans were performed a minimum of four hours after the first milking or after ewe and lamb separation at L69 to allow the udder to accumulate milk [10,12,13] and to have a consistent point in time. Ultrasound scans were undertaken with ewe lambs in a sitting position for easier access to the udder. Ultrasound scans were performed with an ultrasound scanner fitted with a linear transducer with an imaging frequency of 5 to 10 MHz (Mindray Digital Ultrasonic Diagnostic Imaging System DP6600 with 75L38EA, ShenZhen, China). The transducer was applied to the external base of each teat at a 30° angle from the caudal–cranial axis with an inclination of approximately 45° in relation to the teat [10,21]. A light and consistent pressure was applied to the mammary gland through the transducer to minimise the variations related to pressure on the images.

The images recorded included the gland cistern, mammary parenchyma, fat pad and the delimitation between the mammary gland and the abdominal wall. One image of suitable resolution per udder half, where all structures were present, was selected for image processing. Udder halves which had dried off naturally, or that had a palpation score of 4 or 5 and thus considered “abnormal” [19], or identified with mastitis at any time point (P110, W3, W5, W7, L69) were not included in the image selection. Images were discarded for both halves of 2 ewe lambs and one half of 3 ewe lambs.

This method relied on the ability of the operator to interpret and identify lines on the images. The drawing templates of the ultrasound images, created for each time point of Haslin et al. [10], were therefore used to standardize the assessment of each compartment depth. Image processing was undertaken using ImageJ software [22]. The total depth of mammary gland conservative (MTc) and generous (MTg) were the smallest and the largest likely demarcations of the mammary gland visible on the image, respectively [10,23]. The MTc, MTg, fat pad (FP), parenchyma (PAR) and gland cistern (GC) depth were estimated, in millimetres, at the widest point for each subcompartment using the straight tracer. 

#### 2.2.3. Ewe Lamb Milking

Milking used the “oxytocin method” first described by McCance and Alexander [24]. To enable milk let-down, ewe lambs were given 1 IU of synthetic oxytocin (Oxytocin V, 10 IU/mL, PhoenixPharm, Auckland, New Zealand) intravenously. Ewe lambs were then milked in the morning by machine followed by hand milking to empty the udder. The time of the first milking was recorded. The milking procedure was repeated after a minimum of five hours, where the time and milk weight from each udder half were recorded. Daily milk yield per udder half and total daily milk yield were calculated using the following formula [25]:

Daily milk yield = (24 h/Time between milkings) × Milk weight at 2nd milking

Lambs were separated from the ewe lambs, and bottle fed as required, during the 5-h period and reunited after the second milking.

#### 2.2.4. Lamb Measurements

Lambs were ear tagged within 18 h of birth (during twice daily lambing rounds at approximately 11 am and 5 pm) at which time their date of birth, sex, dam ear tag number and birth weights were recorded. Lambs were weighed on each milking day at W3 (approximately 17 days of age), W5 (approximately 31 days of age) and W7 (approximately 44 days of age) between the morning and afternoon milking and again at weaning (L69).

### 2.3. Statistical Analysis

Statistical analyses were conducted with SAS v9.4 (SAS Institute Inc., Cary, NC, USA) and RStudio v1.2. (RStudio Team, PBC, Boston, MA, USA). The final dataset included 30 ewe lambs, their single lambs, and a total of 286 images.

Udder and teat palpations from each udder half (right and left) and udder depth score were analysed with SAS v9.4 using generalised linear models allowing for repeated measurements and assuming Poisson distributions and log transformations. The models for udder and teat palpations included udder half (right vs. left), time point (P110 and L69) and their two-way interaction as fixed effects, and lambing date as a covariate. The model for udder depth score included time point as a fixed effect and lambing date as a covariate. Udder circumference (UC) and UV per udder, and UH, GC, PAR, FP, MTc, MTg and milk yield of each udder half were analysed using general linear models allowing for repeated measurements. The models for UC and UV included time point as a fixed effect and lambing date as a covariate. The models for UH, GC, PAR, FP, MTc, MTg and milk yield included time point (P110, W3, W5, W7, L69) and a two-way interaction between udder half and time point as fixed effects, lambing date as a covariate, and ewe lamb as a random effect.

The residuals of GC, PAR, FP, MTc, MTg and milk yield of both udder halves at P110, W3, W5, W7 and L69 were generated using general mixed models with SAS v9.4 as undertaken by Haslin et al. [10]. The gland cistern (GC), PAR, FP and MTc were adjusted for MTg and lambing date. Udder height (UH), MTg and milk yield per udder half were adjusted for lambing date. Pearson correlations were then used to test for linear associations between time points (P110, W3, W5, W7 and L69) for the residuals of each ultrasound measure (GC, PAR, FP, MTc and MTg) of each udder half with SAS v9.4. Pearson correlations were also used to test for linear associations between the residuals of daily milk yield at W3, W5 and W7 and ultrasound measurements (GC, PAR, FP, MTc and MTg) of each udder half at each time point (P110, W3, W5, W7 and L69).

The residuals of the average of UH, UV, UC, GC, PAR, FP, MTc and MTg of both udder halves at P110, W3, W5, W7 and L69, total daily milk yield at W3, W5 and W7 per udder and lamb growth from birth to W3, birth to W5, birth to W7, W3 to L69, W5 to L69, W7 to L69 and birth to L69 were generated using general mixed models with SAS v9.4 as used by Haslin et al. [10]. Lamb growth, total milk yield, UH, UV, UC and MTg were adjusted for lambing date. Gland cistern (GC), PAR, FP and MTc per udder were adjusted for MTg and lambing date. Pearson correlations were then used to test for associations between the residuals of lamb growth and the residuals of the average of morphological (UH, UC and UV) ultrasound measurements (PAR, FP, GC, MTc and MTg) at each time point and total daily milk yield at W3, W5 and W7.

Multiple regression analyses of daily milk yield at W3, W5 and W7 per udder half were undertaken using general linear mixed models with RStudio v1.2. (Packages “lme4” and “performance”) to enable the calculation of the marginal coefficients of determination [26]. Multiple regression analyses of lamb growth from birth to W3, birth to W5, birth to W7 and birth to L69 were undertaken using general linear models with SAS v9.4. Pearson correlations were used to examine whether each predictive variable was individually correlated with daily milk yield or lamb growth during each period. Predictive variables correlated with daily milk yield or lamb growth with *p* ≤ 0.20 were selected and included in the models [27]. Correlations between selected predictive variables were examined to identify high collinearity (>0.80; [27]). In case of a high collinearity between two predictive variables, only one predictive variable was included in the models based on the biological relevance. Two-way interactions between each of the selected variables were individually tested using general linear models. All non-significant (*p* > 0.05) interactions were excluded from the final model. Backward manual variable eliminations were used to select the model that best explained the variation in daily milk yield and lamb growth by removing predictive variables with *p* > 0.10. Any non-significant (*p* > 0.05) predictive variable causing greater than a 20% change in the model coefficients was considered a confounding variable and included in the models [27]. Confounding effects were evaluated after each variable was removed from the model by checking the changes in predictive variable coefficients. The random effect of ewe lamb was included in the multiple regression models of daily milk yield per udder half. The marginal coefficients of determination of the multiple regressions of daily milk yield per udder half were calculated based on the method of Nakagawa and Schielzeth [26] and corresponded to the variance explained by the selected predictive variables in the final models.

## 3. Results

### 3.1. Udder-Half Differences and Changes Over Time

Udder and teat palpations, UH, GC, PAR, FP, MTc and MTg did not differ between udder halves (*p* > 0.10; data not shown). Udder palpation scores did not differ (*p* > 0.10) between P110 and L69 (Table 2); however, teat palpation scores, UH, UC and UV had lower values (*p* < 0.001) at P110 than at L69 (Table 2). Udder depth scores were greater (*p* < 0.001) at P110 than at L69.

**Figure 1:**
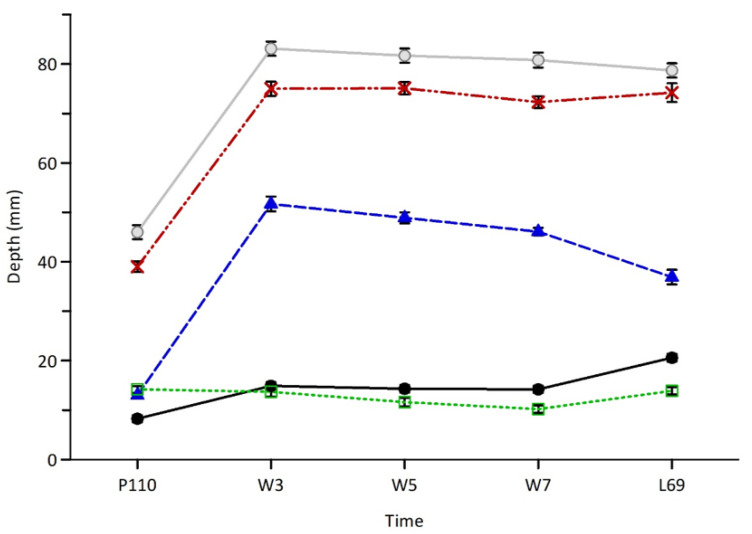
Average depths (±s.e.m.) of the mammary gland cistern (GC; black circles—solid line), mammary fat pad (FP; empty squares—green dotted line), mammary parenchyma (PAR; blue triangles—blue dashed line), total depth of the mammary gland conservative (MTc; red crosses—dashed and dotted line) and generous (MTg; grey circles—solid line) in late pregnancy (P110), lactation (W3, W5, W7) and at weaning (L69). Within lines, averages with different letters were significantly different (*p* < 0.05).

The depth of GC was smaller (*p* < 0.001) at P110 than at W3, W5, W7 and L69, but was greater (*p* < 0.001) at L69 compared to W3, W5, W7, which did not differ (*p* < 0.05; Figure 1). Ewe lambs had a deeper (*p* < 0.05) PAR at W3 and W5 compared to W7, which was greater than L69, which in turn was greater than P110 (Figure 1). The depth of FP was lower (*p* < 0.05) at W7 than P110 with all other points being intermediate (*p* > 0.10; Figure 1). The total depths of the mammary gland (MTc and MTg) were lower (*p* < 0.01) at P110 than MTc and MTg at W3, W5, W7 and L69.

The daily milk yield of the right udder half was greater than the left udder half at W3 (*p* < 0.01; Table 3). The daily milk yield of the left udder half did not differ (*p* > 0.10) between W3 and W5 but was lower (*p* < 0.05) at W7 than at W3 and W5 (Table 3). The daily milk yield of the right udder half and the total daily milk yield of the ewe lamb was greater (*p* < 0.01) at W3 than at W5 and W7 and, was greater at W5 than at W7.

### 3.2. Correlations between Ultrasound Measurements per Udder Half between Time Points

The depth of GC per udder half at W3, W5, W7 and L69 were all positively correlated (*p* < 0.05) but at P110, GC showed no significant (*p* > 0.05) correlations (Table 4). MTg at W3, W5 and W7 were positively correlated (*p* < 0.05) with MTg at L69 (Table 4). The depth of PAR per udder half at P110 was positively correlated (*p* < 0.05) with PAR at W7 (r = 0.302; Appendix A). No other significant associations (*p* > 0.05) were found in PAR per udder half between time points (Appendix A). No significant correlations (*p* > 0.05) were observed between time points for FP (Appendix A). At P110, MTc per udder half was positively correlated with MTc at W7 (*p* < 0.001; r = 0.459), but no other significant correlations (*p* > 0.05) were observed between time points for MTc (Appendix A, Table A1). 

### 3.3. Associations of Daily Milk Yield and Ultrasound Measurements per Udder Half

Daily milk yield per udder half at W3 was positively associated (*p* < 0.05) with GC at P110 and at W3 (Table 5) but was negatively associated with FP at P110 per udder half (*p* < 0.001; Table 5). Daily milk yield at W5 was positively correlated (*p* < 0.05) with MTg at W3 and W5 per udder half but was negatively correlated (*p* < 0.001) with GC at L69 (Table 5). Daily milk yield at W7 per udder half was positively associated with FP at P110 (*p* < 0.05), MTg at W3 and at L69, but was negatively associated (*p* < 0.05) with GC at L69 (Table 5). Correlations that were not significant (*p* > 0.05) are presented in Appendix A, Table A2.

### 3.4. Prediction of Daily Milk Yield per Udder Half Using Udder Measurements

The best regression model for milk yield at W3 per udder half explained 26.8% of the variation and included the effects of GC, PAR, FP at P110, GC and MTg at W3 (Table 6). An average ewe lamb had a gland cistern of 8.24 mm and 14.95 mm and a ewe lamb in the 90th percentile had a gland cistern of 10.9 mm and 23.0 mm at P110 and W3 resulting in 48 g/d and 125 g/d difference in milk yield per udder half at week three of lactation, respectively. The best regression model for milk yield at W5 per udder half explained 21.4% of the variation and included the effects of MTc at W3, PAR, MTc at W5 and the interactions between PAR at W5 and MTc at W3, and MTc at W3 and MTc at W5 (Table 6). The best regression model for milk yield at W7 per udder half explained 38.4% of the variation and included the effects of FP, UH at P110, MTg at W3, MTc at W7 and the interactions between MTg at W3 and UH at P110, and MTc at W7 and UH at P110 (Table 6).

### 3.5. Associations between Lamb Growth and Udder Measurements and Milk Yield per Udder

Lamb growth from birth to W3, birth to W5, birth to W7 and birth to weaning (L69) were positively correlated (*p* < 0.05) with total daily MY at W3, W5 and W7 (Table 7). Lamb growth from W3 to L69 and W5 to L69 were positively correlated (*p* < 0.05) with total daily milk yield at W7 (Table 7).

Lamb growth from birth to W3 was negatively correlated (*p* < 0.05) with the average FP of both udder halves at P110 and W3 (Table 7). Lamb growth from birth to W5 was negatively correlated (*p* < 0.01) with the average FP of both udder halves at W3 (Table 7). Lamb growth from W3 to L69 and birth to L69 were positively correlated (*p* < 0.05) with UC and UV at L69 (Table 7). Lamb growth from W7 to L69 was negatively correlated with PAR at W3 (*p* < 0.05; Table 7). Non-significant correlations (*p* > 0.05) between udder measurements and lamb growth are presented in Appendix A, Table A3.

### 3.6. Predictions of Lamb Growth Using Ultrasound Measurements

The best regression model for lamb growth from birth to W3 explained 63.5% of the variation and included the effect of the average of GC, FP at P110, FP, PAR and MTc at W3 of both udder halves, and the interaction between PAR and MTc at W3 (Table 8). An average ewe lamb had a fat pad of 14.2 mm and a ewe lamb with a larger fat pad, in the 90th percentile, had a fat pad of 19.0 mm at P110 resulting in 41.7 g/d in difference in single lamb growth from birth to W3. An average ewe lamb had a parenchymal depth of 51.8 mm and a ewe lamb in the 90th percentile had a parenchymal depth of 61.8 mm at W3 resulting in 55.4 g/d in difference in single lamb growth from birth to W3. Prediction of single lamb growth from birth to W5 was not significantly (*p* > 0.05) predicted by ultrasound and morphological measurements per udder (Table 8). The best regression model for lamb growth from birth to W7 explained 38.0% of the variation and included the effect of the average of PAR, MTc at W3 of both udder halves, and the interaction between PAR and MTc at W3 (Table 8). The best regression model for lamb growth from birth to weaning (L69) explained 36.4% of the variation and included the effect of the average of MTc at W3, FP at W7 and at L69 of both udder halves (Table 8).

## 4. Discussion

### 4.1. Prediction of Milk Yield Using Ultrasound and Morphological Measurements

The milk yield of ewe lambs in this experiment decreased over time, following the normal progress of lactation [28]. In the present experiment, milk yield differed between the right and left udder halves at week three of lactation (i.e., near the peak of lactation [29]). This difference in production may be explained by a preference of the single lamb for one udder half over the other, which may have resulted in an overstimulation of this udder half [30]. This overstimulation would lead to an increase in milk production to adapt to the demand of the offspring [29]. The difference between udder halves, however, did not persist over time.

It was hypothesised that the cistern depth of ewe lamb mammary glands would be positively associated with milk yield, as reported in mature dairy ewes. While this was the case, the predictions of milk yield at three, five and seven weeks of lactation from udder ultrasound and morphological measurements were moderate (21, 27 and 38%). This finding was greater than that of van der Linden et al. [16] who reported that udder dimensions explained 19% of the variation in milk yield at day 21 and 28 of lactation. The maximum variation explained for milk yield at week seven of lactation using udder measurements was moderate (38%). van der Linden et al. [16] explained a maximum of 36% of the variation in milk yield at day 35 of lactation using udder dimensions. Arcos-Álvarez et al. [31] and Espinosa-Mendoza et al. [32] explained 54 to 63% of the variation in milk yield using udder dimensions. In dairy heifers, the proportion of secretory tissue in the parenchyma measured 15 to seven days prior to calving was highly positively correlated (0.80) with milk yield over 100 days of lactation, indicating that this measure was an accurate indicator of milk yield [14]. In these studies, milk yield was predicted using udder dimensions [16,31,32] or ultrasound measurements [14] only, whereas in the current experiment, milk yield was predicted using both udder dimensions and their internal structures and this may explain some of the differences observed. The difference between our data and those of Strzetelski et al. [14] is likely due to the difference in species and their purpose (dairy heifers vs. non-dairy ewe lambs). Milk production is a complex biological process primarily determined by the number of secretory cells and their activity [33,34,35,36]. While ultrasound imaging enables the visualisation and assessment of the dimensions of the different tissues in the mammary gland [23,37] and their echo-textural characteristics [2,38], it does not provide information on the number and activities of secretory cells. This may explain the moderate prediction of milk yield using ultrasound measurements in the current experiment and is a limitation to the use of ultrasound as a technique to predict milk yield. Further research is needed to identify more accurate indicators of milk yield such as gene expression in ewe mammary gland.

Milk yield at week three and five of lactation were only usefully predicted by ultrasound measurements, whereas, at week seven, milk yield was predicted using both ultrasound and morphological measurements. Positive relationships between udder morphological measurements and milk yield have been well documented in dairy ewes [39]. Milk yields were, however, better predicted using udder ultrasound measurements in late pregnancy and early lactation. Udder measurements collected prior to lambing (i.e., in late pregnancy) may enable farmers to identify ewe lambs that may have greater milk yield in lactation earlier and potentially select these ewe lambs.

Mammary gland cistern depth in late pregnancy and early lactation were positively associated with milk yield per udder half in week three of lactation. These findings are consistent with previous studies that reported that mature ewes with larger cisterns produced more milk than ewes with smaller cisterns [8,11,13]. This is perhaps an unsurprising result as the mammary gland cistern is the cavity where milk is stored between suckling events or milking [34]. Larger cisterns, therefore, enable greater storage capacity for milk until removal [9]. The contribution of gland cistern depth in late pregnancy and early lactation to the predicted milk production at week three of lactation, however, was moderate. For example, there were differences of 48 and 125 g/d of milk per udder half during week three of lactation between a ewe lamb with an average cistern depth and those with a larger cistern in late pregnancy and early lactation, respectively. Assuming that milk production is constant during the third week of lactation, these differences would result in an increase of 336 and 875 g of milk per udder half and a total increase of 672 g and 1750 g of milk per ewe lamb during week three of lactation, respectively. Danso et al. [40] reported that for 1 kg of milk, lamb growth would increase 130 g between birth and 42 days of age. Hence, using the data of Danso et al. [40], the difference in milk yield at week three of lactation in the current experiment would lead to an increase of 87 g and 228 g in lamb growth born to ewes with larger gland cistern in late pregnancy and early lactation, respectively. Although, the depths of the gland cistern in pregnancy and early lactation were positively associated with milk yield, the ultrasound method would not be an accurate technique to identify ewe lambs that would have greater milk production.

### 4.2. Prediction of Lamb Growth Rates Using Ultrasound Measurements

The prediction of lamb growth to weaning using morphological and ultrasound measurements was moderate (36%); however, lamb growth rates between birth and week three of lactation were better predicted (64%). The percentage of the variation in lamb growth between birth and weaning explained by the model was consistent with that reported by Haslin et al. [10] (37%). During the first three to four weeks of life, lambs are solely dependent on milk to survive [41,42]. This early reliance of milk likely explains the greater proportion of variation in lamb growth to week three of lactation being explained by udder measurements. In addition, lamb growth was poorly to moderately predicted by milk yield [16,40]. The proportion of variation explained for lamb growth during early lactation in the current experiment was greater than that found by Haslin et al. [10] (12%). In the present experiment, the lambs were approximately 17 days of age when lactation measurements were recorded, whereas the lambs of Haslin et al. [10] were 29 days of age and, therefore, likely to be less dependent on milk. Regardless of these differences, the models predicted a limited amount of the variation in lamb growth, particularly between birth and weaning. Lamb growth depends on multiple factors, including milk yield and quality [43,44], and the quantity and quality of solid feed available [42]. It is likely, therefore, that ultrasound and morphological measurements of the udder alone may not explain enough variation in lamb growth to be accurate predictors. Further research is required to identify more accurate indicators of lamb growth.

Lamb growth from birth to week three of lactation was predicted by the ultrasound measurements of the fat pad and gland cistern in late pregnancy and of parenchyma and fat pad in early lactation. These findings contrast with those of Haslin et al. [10], in ewe lambs, where the depth of the gland cistern in early lactation was the only predictor of lamb growth to early lactation. In the current experiment, the ultrasound measurements that predicted lamb growth to weaning included measurements in week three and seven of lactation and weaning. This finding also contrasts with the results of Haslin et al. [10] who reported that lamb growth to weaning was predicted by the depth of the parenchyma in late pregnancy and gland cistern depth in early lactation. Mammary gland predictors of lamb growth measured in late pregnancy may enable an early identification of ewe lambs that would have lambs with greater growth rates. More research, however, is needed to determine whether ewe lambs with larger mammary gland cisterns or parenchyma would have similar mammary characteristics in subsequent years and, therefore, whether ultrasound could be used to select ewe lambs with superior lactation performance.

The depth of the fat pad in late pregnancy was positively correlated with lamb growth to week three of lactation. The contribution of fat pad depth in late pregnancy to the model was moderate, with singletons born to ewe lambs with a large fat pad in late pregnancy being 626 and 876 g heavier at 15 and 21 days of age, respectively, compared to single lambs born to ewe lambs with an average fat pad. Single lambs born to ewes with large fats pad in late pregnancy would be predicted to be heavier in early lactation when they will be yarded for the first time (approximately 30 days of age) than single lambs born to ewes with an average fat pad. The fat pad is predominantly composed of adipose and connective tissue [45,46]. It has been reported that the amount of adipose tissue in the fat pad dictates the total number of secretory cells [45,47], which then determines milk production [35]. The fat pad also has local-synthesized IGF-1, which stimulates the growth of mammary parenchyma [46]. The fat pad is also involved in lipid storage during pregnancy and supports milk production in lactation with the biosynthesis of lipids [45]. Thus, it is perhaps not surprising that a deeper fat pad in late pregnancy was linked with greater single lamb growth rates.

The depth of the parenchyma at week three of lactation was moderately and positively associated with lamb growth to week three of lactation. Single lambs born to ewe lambs with a large parenchyma at week three of lactation were 831 and 1163 g heavier at 15 and 21 days of age, respectively, than single lambs born to ewe lambs with an average parenchyma depth. Single lambs born to ewe lambs with large parenchyma depth at week three of lactation would be predicted to be heavier by more than 1 kg in early lactation when they will be yarded for the first time (approximately 30 days of age) than single lambs born to ewe lambs with an average parenchyma depth. The cells involved in milk production and secretion are located in the mammary parenchyma [29,48] and their number and activity determine the quantity of milk produced [33,35]. The development of the parenchyma primarily occurs in late pregnancy (day 110 to 141 of pregnancy; [29,49]), thus, a larger parenchyma at week three of lactation may indicate a greater number of secretory cells and possibly greater milk production. The ultrasound method used in the current experiment, therefore, was a potential technique that could be used to determine mammary parenchyma tissue depth as an indicator of early lamb growth.

Among ewe lambs [10], the depth of the mammary parenchyma in late pregnancy and the gland cistern in early lactation were indicators of single lamb growth to weaning. In this experiment, however, only the total depth of the mammary gland conservative at week three of lactation and the depth of the fat pad at week seven of lactation and at weaning were predictors of lamb growth to weaning. The difference in the predictors of lamb growth could be due to differences in the timing of the measurements. Parenchymal tissue development primarily occurs between day 110 and 141 of pregnancy [29,50]. The ultrasound measurements made in late pregnancy were recorded between 102 and 122 days of pregnancy whereas in Haslin et al. [10], they were recorded between 100 and 115 days of pregnancy. This small difference in timing may explain why the depth of parenchyma in late pregnancy was not a good indicator of lamb growth to weaning in the current experiment. Further research, using a greater number of ewe lambs, is warranted to better understand the indicators of single lamb growth to weaning using ultrasound measurements of the internal structure of ewe lamb mammary gland. This knowledge would determine if ultrasound could be used as an early technique to identify ewe lambs with superior characteristics for increased growth of their single lambs.

## 5. Conclusions

Although the mammary gland cistern depths in late pregnancy and third week of lactation were identified as indicators of non-dairy ewe lamb milk yield in early lactation, the prediction of milk yield using ultrasound measurements was poor. The prediction of the growth of single lambs from birth to the third week of lactation using udder measurements was high, whereas the prediction of growth to weaning of single lambs was moderate. The size of the mammary fat pad in late pregnancy and the parenchyma in early lactation were indicators of lamb growth to week three of life. This ultrasound method was not an accurate technique for predicting milk yield in non-dairy ewe lambs; however, it could potentially provide farmers with a technique for an early selection of non-dairy Romney ewe lambs whose progeny would have faster early growth rates. More research is warranted to find more accurate indicators of milk yield and to determine whether ultrasound could be used as an accurate technique to identify and select ewe lambs that would have greater single lamb growth rates to weaning.

## Figures and Tables

**Table 1:** Description of the milking groups created based on parturition dates in order to milk all ewe lambs in week three (W3), five (W5) and seven (W7) of lactation.

Milking Group	N	Lambing Dates	Days of Lactation at Milking		
			W3	W5	W7
1	7	27/09–4/10	16	30	44
2	15	5/10–11/10	15	29	43
3	21	12/10–21/10	18	32	46

**Table 2:** Effect of time (day 110 of pregnancy (P110) and weaning of the lambs (L69)) on udder and teat palpations, udder depth score, udder height (UH), circumference (UC), volume (UV). Least square means ± s.e.m.

Parameters	P110	L69
Udder palpation	1.23 ± 0.11	1.57 ± 0.17
Teat palpation	1.0 ± 0 ^a^	1.18 ± 0.07 ^b^
Udder depth score	4.93 ± 0.05 ^b^	3.69 ± 0.06 ^a^
UH (cm)	4.90 ± 0.22 ^a^	10.3 ± 0.21 ^b^
UC (cm)	26.1 ± 0.39 ^a^	40.8 ± 0.96 ^b^
UV (cm^3^)	270.3 ± 13.4 ^a^	1393 ± 83.9 ^b^

^a,b^ Within rows, means with different superscripts are significantly different (*p* < 0.05).

**Table 3:** Effect of time of lactation (week 3 (W3), week 5 (W5) and week 7 (W7)) on daily milk yield of the right and left udder half and total daily milk yield of ewe lambs. Least square means ± s.e.m.

Milk Yield (g/d)	W3	W5	W7
Left udder half	910 ± 39.4 ** ^b^	876 ± 39.6 ^b^	723 ± 39.4 ^a^
Right udder half	1076 ± 38.4 ** ^c^	927 ± 38.9 ^b^	749 ±38.4 ^a^
Total milk yield	1924 ± 65.3 ^c^	1740 ± 66.1 ^b^	1421 ± 65.3 ^a^

** Daily milk yield of the right udder half differed from milk yield of the left udder half (*p* < 0.01); ^a,b,c^ Within rows, means with different superscripts are significantly different (*p* < 0.05).

**Table 4:** Correlation coefficients of residuals of depths of the mammary gland cistern (GC) and total depth of mammary gland generous (MTg) per udder half in late pregnancy (P110), week 3 (W3), week 5 (W5), week 7 (W7) of lactation and at weaning (L69).

Parameters	W3	W5	W7	L69
GC				
W3		0.398 ***	0.257 *	0.336 *
W5			0.534 ***	0.504 ***
W7				0.373 **
MTg				
W3		0.193	0.026	0.546 ***
W5			−0.082	0.297 *
W7				0.333 *

* *p* < 0.05; ** *p* < 0.01; *** *p* < 0.001.

**Table 5:** Correlation coefficients of residuals of daily milk yield in week 3 (W3), week 5 (W5), week 7 (W7) of lactation, gland cistern (GC) at P110, W3 and L69, fat pad (FP) at P110 and total depth of the mammary gland generous (MTg) at W3, W5 and L69 per udder half.

Parameters	Milk Yield W3	Milk Yield W5	Milk Yield W7
GC			
P110	0.314 **	0.077	−0.120
W3	0.288 *	−0.003	−0.018
L69	−0.030	−0.499 ***	−0.326 *
FP			
P110	−0.409 ***	−0.053	0.325 *
MTg			
W3	0.175	0.327 **	0.277 *
W5	0.175	0.329 **	0.230
L69	0.129	0.178	0.352 *

* *p* < 0.05; ** *p* < 0.01; *** *p* < 0.001; P110: day 110 of pregnancy; L69: weaning.

**Table 6:** Multiple regression coefficients (±s.e.m.) of ultrasound (GC, PAR, FP, MTc and MTg) and morphological measurements (UH) in pregnancy (P110), week 3 (W3), 5 (W5) and 7 (W7) of lactation on daily milk yield (MY) in week 3, 5 and 7 of lactation per udder halves (g/d).

Independent Variables Selected	Daily MY at W3 R^2^ = 0.268	Daily MY at W5 R^2^ = 0.214	Daily MY at W7 R^2^ = 0.384
Intercept	770 ± 302	4966 ± 2358	−5666 ± 1606
GC (P110)	18.1 ± 9.3 ^1^	-	-
PAR (P110)	−9.2 ± 10.2	-	-
FP (P110)	−13.1 ± 8.2	-	12.0 ± 4.77
UH (P110)	- ^3^	-	1146 ± 325
GC (W3)	15.5 ± 7.2	-	-
MTc (W3)	-	−65.3 ± 31.5	-
MTg (W3)	1.8 ± 3.2	-	41.4 ± 13.1
MTg (W3) × UH (P110) ^2^	-	-	−7.2 ± 2.59
PAR (W5)	-	38.0 ± 26.4	-
MTc (W5)	-	−80.0 ± 32.0	-
PAR (W5) × MTc (W3)	-	−0.46 ± 0.36	-
MTc (W3) × MTc (W5)	-	1.18 ± 0.44	-
MTc (W7)	-	-	40.8 ± 16.0
MTc (W7) × UH (P110)	-	-	−8.0 ± 3.38
Ewe	14,238 ± 11,175	14,118 ± 13,208	1112 ± 4182

MY: Milk yield; GC: Gland cistern; PAR: Parenchyma; FP: Fat Pad; UH: Udder height; MTg: Total mammary depth generous; MTc: Total mammary depth conservative. ^1^ For each 1 mm increase in depth of the gland cistern (GC) in pregnancy (P110), daily milk yield at week 3 of lactation (MY at W3) increased by 18.1 ± 9.3 g/d; ^2^ Two-way interaction between MTg at W3 and UH at P110; ^3^ Independent variable that was not a significant (*p* > 0.05) predictor of daily milk yield at W3.

**Table 7:** Correlation coefficients of residuals of lamb growth from birth to week 3 (Birth to W3), birth to week 5 (Birth to W5), birth to week 7 of lactation (Birth to W7), W3 to weaning (W3 to L69), W5 to weaning (W5 to L69), W7 to weaning (W7 to L69), birth to weaning (Birth to L69), total daily milk yield in W3, W5 and W7, udder circumference (UC), udder volume (UV) at weaning (L69), the average of both udder halves of parenchyma (PAR) at W3 and fat pad (FP) at P110 and W3.

Parameters	Birth to W3	Birth to W5	Birth to W7	W3 to L69	W5 to L69	W7 to L69	Birth to L69
Milk yield							
W3	0.711 ***	0.631 ***	0.579 ***	0.181	0.115	0.033	0.532 **
W5	0.500 **	0.528 **	0.477 **	0.168	0.096	0.128	0.466 *
W7	0.367 *	0.480 **	0.628 ***	0.526 **	0.523 **	0.296	0.633 ***
UC							
L69	0.057	0.222	0.349	0.410 *	0.269	0.200	0.454 *
UV							
L69	0.069	0.190	0.315	0.422 *	0.336	0.303	0.455 *
PAR							
W3	0.326	0.141	0.103	−0.227	−0.191	−0.378 *	−0.037
FP							
P110	−0.370 *	−0.299	−0.217	0.059	0.148	0.242	−0.133
W3	−0.592 **	−0.534 **	−0.369	0.081	0.217	0.397	−0.220

* *p* < 0.05; ** *p* < 0.01; *** *p* < 0.001. P110: day 110 of pregnancy; L69: weaning.

**Table 8:** Multiple regression coefficients (±s.e.m.) of the average of ultrasound (GC, PAR, FP and MTc) measurements in pregnancy (P110), week 3 (W3), 5 (W5) and 7 (W7) of lactation on single lamb growth from birth to week 3 (Birth to W3), birth to week 5 (Birth to W5), birth to week 7 of lactation (Birth to W7) and birth to weaning (Birth to L69).

Lamb Growth	Independent Variables Selected									
(g/d)	Intercept	GC (P110)	FP (P110)	FP (W3)	PAR (W3)	PAR × MTc (W3) ^2^	MTc (W3)	FP (W7)	FP (L69)	R ^2^
Birth to W3	−43.5 ± 1213	−3.6 ± 6.0 ^1^	8.11 ± 5.6	−8.66 ± 2.87	5.72 ± 24	−6.7 × 10^−3^ ± 0.31	1.17 ± 15	- ^3^	-	0.635
Birth to W5	NS	-	-	-	-	-	-	-	-	-
Birth to W7	−1165 ± 524	-	-	-	25.6 ± 10.3	−0.31 ± 0.13	18.1 ± 0.67	-	-	0.380
Birth to L69	−52.9 ± 101	-	-	-	-	-	4.45 ± 1.40	−1.14 ± 2.57	2.16 ± 2.11	0.364

GC: Gland cistern; FP: Fat pad; PAR: Parenchyma; MTc: Total mammary depth conservative. ^1^ For each 1 mm increase in depth of the gland cistern in pregnancy (P110), lamb growth decreased by 3.6 ± 6.0 g/d from birth to week 3 of lactation (Birth to W3); ^2^ Two-way interaction between PAR at W3 and MTc at W3; ^3^ Independent variable that was not a significant (*p* > 0.05) predictor of lamb growth from birth to W3.

## Data Availability

The data presented in this experiment are available within the article.

## Appendix A. Correlations among Udder Measurements, Milk Yield and Lamb Growth

**Table A1:** Correlation coefficients of residuals of depths of the mammary parenchyma (PAR), fat pad (FP) and total depth of mammary gland conservative (MTc) per udder half in pregnancy (P110), week 3 (W3), week 5 (W5), week 7 (W7) of lactation and at weaning (L69).

Parameters	W3	W5	W7	L69
PAR				
P110	0.196	−0.046	0.302 *	−0.068
W3		0.094	−0.017	−0.098
W5			0.085	0.028
W7				0.173
GC				
P110	0.011	−0.058	−0.089	0.102
FP				
P110	0.376 *	−0.126	0.141	−0.038
W3		0.113	0.193	0.036
W5			−0.204	0.276
W7				0.153
MTc				
P110	0.021	0.004	0.459 *	0.063
W3		−0.033	−0.062	−0.218
W5			−0.179	0.200
W7				0.203
MTg				
P110	0.238	−0.031	0.195	0.265

* *p* < 0.05.

**Table A2:** Correlation coefficients of residuals of daily milk yield in week 3 (W3), week 5 (W5), week 7 (W7) of lactation, udder height (UH) in pregnancy (P110) and at weaning (L69), gland cistern (GC), parenchyma (PAR), fat pad (FP), total depth of the mammary gland conservative (MTc) and total depth of the mammary gland generous (MTg) at P110, W3, W5, W7, L69 per udder half.

Parameters	Milk Yield W3	Milk Yield W5	Milk Yield W7
UH			
P110	0.013	−0.186	−0.256
L69	−0.006	0.116	0.143
GC			
W5	−0.008	−0.141	−0.065
W7	−0.014	−0.080	0.040
PAR			
P110	−0.172	−0.016	−0.167
W3	0.014	−0.036	−0.048
W5	0.028	0.220	0.011
W7	−0.016	0.113	−0.030
L69	0.026	0.121	0.185
FP			
W3	−0.112	−0.047	0.297
W5	0.057	−0.128	−0.199
W7	−0.046	0.073	0.031
L69	−0.0007	0.273	0.028
MTc			
P110	−0.170	−0.133	0.037
W3	−0.055	−0.129	−0.131
W5	0.052	−0.047	−0.101
W7	−0.129	−0.029	−0.008
L69	0.022	0.104	0.147
MTg			
P110	0.119	−0.107	0.120
W7	0.209	0.002	0.219

P110: day 110 of pregnancy; L69: weaning.

**Table A3:** Correlation coefficients of residuals of lamb growth from birth to week 3 of lactation (Birth to W3), birth to week 5 of lactation (Birth to W5), birth to week 7 of lactation (Birth to W7), L3 to weaning (W3 to L69), W5 to weaning (W5 to L69), W7 to weaning (W7 to L69), birth to weaning (Birth to L69), the average of both udder half of udder height (UH) in pregnancy (P110) and at weaning (L69), udder circumference and volume at P110, the average of both udder halves of gland cistern (GC), parenchyma (PAR) and fat pad at P110, W3, W5, W7, L69.

Parameters	Birth to W3	Birth to W5	Birth to W7	W3 to L69	W5 to L69	W7 to L69	Birth to L69
UH							
P110	−0.043	−0.280	−0.269	−0.138	0.017	0.117	−0.167
L69	0.031	0.015	0.040	0.134	0.139	0.222	0.152
UC							
P110	−0.172	−0.171	−0.053	0.088	0.130	0.048	−0.021
UV							
P110	−0.085	−0.234	−0.162	−0.020	0.092	0.100	−0.080
GC							
P110	0.290	0.134	0.082	−0.007	0.018	0.091	0.147
W3	−0.085	0.093	0.090	0.168	0.063	0.113	0.117
W5	−0.126	0.067	0.126	0.253	0.158	0.062	0.126
W7	0.031	0.126	0.133	0.040	−0.076	−0.154	0.051
L69	−0.057	−0.045	−0.043	−0.004	−0.053	−0.086	−0.042
PAR							
P110	−0.008	−0.127	−0.160	−0.209	−0.133	−0.120	−0.175
W5	0.098	−0.009	−0.028	−0.031	0.041	0.163	0.049
W7	−0.012	−0.167	−0.282	−0.302	−0.248	−0.079	−0.259
L69	V0.081	−0.077	−0.061	−0.068	−0.071	−0.107	−0.087
FP							
W5	0.012	−0.090	−0.119	−0.120	−0.132	−0.118	−0.117
W7	0.090	0.171	0.228	0.265	0.259	0.262	0.291
L69	0.126	0.257	0.275	0.267	0.228	0.235	0.287

P110: day 110 of pregnancy; L69: weaning.

## References

[1] Petridis I.G., Gouletsou P.G., Barbagianni M.S., Amiridis G.S., Brozos C., Valasi I., Fthenakis G.C. (2014). Ultrasonographic findings in the ovine udder during involution. J. Dairy Res..

[2] Barbagianni M.S., Gouletsou P.G., Valasi I., Petridis I.G., Giannenas I., Fthenakis G.C. (2015). Ultrasonographic findings in the ovine udder during lactogenesis in healthy ewes or ewes with pregnancy toxaemia. J. Dairy Res..

[3] Barbagianni M.S., Mavrogianni V.S., Vasileiou N.G.C., Fthenakis G.C., Petridis I.G. (2017). Ultrasonographic examination of the udder in sheep. Small Rumin. Res..

[4] Makovický P., Makovický P., Nagy M., Milerski M., Margetín M. (2019). Use of ultrasonography for determination of cistern size in different genotypes of dairy sheep. Pol. J. Nat. Sci..

[5] Makovický P., Nagy M., Margetín M., Poráčová J., Milerski M., Makovický P. (2019). Measurement of the Mammary Gland Cistern of Dairy Ewes. Acta Univ. Agric. Et Silvic. Mendel. Brun..

[6] Franz S., Hofmann-Parisot M., Gütler S., Baumgartner W. (2003). Clinical and ultrasonographic findings in the mammary gland of sheep. N. Z. Vet. J..

[7] Makovicky P., Milerski M., Margetín M. (2017). B-mode Ultrasonography of Mammary Glands in Dairy Ewes during the Lactation Period. Rev. Científica.

[8] Nudda A., Pulina G., Vallebella R., Bencini R., Enne G. (2000). Ultrasound technique for measuring mammary cistern size of dairy ewes. J. Dairy Res..

[9] Castillo V., Such X., Caja G., Salama A.A.K., Albanell E., Casals R. (2008). Changes in alveolar and cisternal compartments induced by milking interval in the udder of dairy ewes. J. Dairy Sci..

[10] Haslin E., Corner-Thomas R.A., Kenyon P.R., Molenaar A.J., Morris S.T., Blair H.T. (2021). Mammary Gland Structures Are Not Affected by an Increased Growth Rate of Yearling Ewes Post-Weaning but Are Associated with Growth Rates of Singletons. Animals.

[11] Rovai M., Caja G., Such X. (2008). Evaluation of udder cisterns and effects on milk yield of dairy ewes. J. Dairy Sci..

[12] Ruberte J., Carretero A., Fernandez M., Navarro M., Caja G., Kirchner F., Such X. (1994). Ultrasound mammography in the lactating ewe and its correspondence to anatomical section. Small Rumin. Res..

[13] Caja G., Such X., Ruberte J., Carretero A., Navarro M. (1999). The use of ultrasonography in the study of mammary gland cisterns during lactation in sheep. Proc. Eur. Soc. Anim. Prod..

[14] Strzetelski J., Bilik K., Niwińska B., Skrzyński G., Łuczyńska E. (2004). Ultrasound evaluation of the mammary gland structure in preparturient heifers vs performance of first calvers. J. Anim. Feed Sci..

[15] Torres-Hernandez G., Hohenboken W. (1980). Relationships between ewe milk production and composition and preweaning lamb weight gain. J. Anim. Sci..

[16] Van der Linden D.S., Lopez-Villalobos N., Kenyon P.R., Thorstensen E., Jenkinson C.M.C., Peterson S.W., Blair H.T. (2010). Comparison of four techniques to estimate milk production in singleton-rearing non-dairy ewes. Small Rumin. Res..

[17] Corner R.A., Blair H.T., Morris S.T., Kenyon P.R. (2013). BRIEF COMMUNICATION: A comparison of aspects of the reproductive success of ewe lamb and mixed age ewes joined over the same period. Proc. N. Z. Soc. Anim. Prod..

[18] Kenyon P.R., Webby R.W., Rattray P.V., Brooks I.M., Nicol A.M. (2017). Pasture and Supplements in Sheep Production Systems. Pasture and Supplements for Grazing Animals.

[19] Griffiths K.J., Ridler A.L., Compton C.W.R., Corner-Thomas R.A., Kenyon P.R. (2019). Investigating associations between lamb survival to weaning and dam udder and teat scores. N. Z. Vet. J..

[20] Ayadi M., Such X., Ezzehizi N., Zouari M., Najar T., M’Rad M.B., Casals R. (2011). Relationship between mammary morphology traits and milk yield of Sicilo-Sarde dairy sheep in Tunisia. Small Rumin. Res..

[21] Albino R.L., Marcondes M.I., Akers R.M., Detmann E., Carvalho B.C., Silva T.E. (2015). Mammary gland development of dairy heifers fed diets containing increasing levels of metabolisable protein: Metabolisable energy. J. Dairy Res..

[22] Ferreira T., Rasband W.S. ImageJ User Guide—IJ 1.46. Imagej.nih.gov/ij/docs/guide/.

[23] Molenaar A.J., Maclean P.H., Gilmour M.L., Draganova I.G., Symes C.W., Margerison J.K., McMahon C.D. (2020). Effect of whole-milk allowance on liveweight gain and growth of parenchyma and fat pads in the mammary glands of dairy heifers at weaning. J. Dairy Sci..

[24] McCance I., Alexander G. (1959). The onset of lactation in the Merino ewe and its modification by nutritional factors. Aust. J. Agric. Res..

[25] Van der Linden D.S., Kenyon P.R., Blair H.T., Lopez-Villalobos N., Jenkinson C.M.C., Peterson S.W., Mackenzie D.D.S. (2009). Effects of ewe size and nutrition on fetal mammary gland development and lactational performance of offspring at their first lactation. J. Anim. Sci..

[26] Nakagawa S., Schielzeth H. (2013). A general and simple method for obtaining R2 from generalized linear mixed-effects models. Methods Ecol. Evol..

[27] Dohoo I.R., Martin W., Stryhn H.E. (2003). Model-Building Strategies. Veterinary Epidemiologic Research.

[28] Cardellino R.A., Benson M.E. (2002). Lactation curves of commercial ewes rearing lambs. J. Anim. Sci..

[29] Lérias J.R., Hernández-Castellano L.E., Suárez-Trujillo A., Castro N., Pourlis A., Almeida A.M. (2014). The mammary gland in small ruminants: Major morphological and functional events underlying milk production–a review. J. Dairy Res..

[30] Makovicky P., Milerski M., Margetín M., Makovicky P., Nagy M. (2015). Genetic parameters for the size of udder cisterns in ewes diagnosed by ultrasonography among breeds: Improved Valachian, Tsigai, Lacaune and their crosses. Arch. De Zootec..

[31] Arcos-Álvarez D., Canul-Solís J., García-Herrera R., Sarmiento-Franco L., Piñeiro-Vazquez Á., Casanova-Lugo F., Tedeschi L.O., Gonzalez-Ronquillo M., Chay-Canul A. (2020). Udder Measurements and Their Relationship with Milk Yield in Pelibuey Ewes. Animals.

[32] Espinosa-Mendoza R.I., Arcos-Álvarez D.N., Garcia-Herrera R.A., Antonio-Molina G., Vicente-Pérez R., Macias-Cruz U., Ronquillo M.G., Chaparro A.C.L., Chay-Canul A.J. (2020). Predicting milk yield in Pelibuey ewes from the udder volume measurement with a simple method. J. Dairy Res..

[33] Capuco A.V., Wood D.L., Baldwin R., Mcleod K., Paape M.J. (2001). Mammary cell number, proliferation, and apoptosis during a bovine lactation: Relation to milk production and effect of bST. J. Dairy Sci..

[34] Akers R.M., Akers R.M. (2002). Chapter 2. Mammary Development, Anatomy, and Physiology. Lactation and the Mammary Gland.

[35] Boutinaud M., Guinard-Flament J. (2004). The number and activity of mammary epithelial cells, determining factors for milk production. Reprod. Nutr. Dev..

[36] Capuco A.V., Akers R.M., Greenwood P.L., Bell A.W., Vercoe P.E., Viljoen G.J. (2010). Management and environmental influences on mammary gland development and milk production. Managing the Prenatal Environment to Enhance Livestock Productivity.

[37] Molenaar A., Leatha S.R., Cajab G., Hendersona H.V., Camerona C., Challiesa M., Taukiria K., Chikazhec T., Kaumoanac S., Lannoua B. (2013). Brief Communication: Development of ultrasound methodology to measure cow udder cistern storage capacity in the New Zealand pasture-fed context. Proc. N. Z. Soc. Anim. Prod..

[38] Murawski M., Schwarz T., Jamieson M., Ahmadi B., Bartlewski P.M. (2019). Echotextural characteristics of the mammary gland during early lactation in two breeds of sheep varying in milk yields. Anim. Reprod..

[39] Pourlis A. (2020). Ovine mammary morphology and associations with milk production, milkability and animal selection. Small Rumin. Res..

[40] Danso A.S., Morel P.C.H., Kenyon P.R., Blair H.T. (2016). Relationships between prenatal ewe traits, milk production, and preweaning performance of twin lambs. J. Anim. Sci..

[41] Geenty K.G., Sykes A.R., Familton A.S. (1983). Feed requirements of the ewe and lamb between birth and weaning. Lamb Growth-Tehnical Handbook.

[42] Danso A.S., Morel P.C.H., Kenyon P.R., Blair H.T. (2014). Brief communication: Effect of early life diet on lamb growth and organ development. Proc. N. Z. Soc. Anim. Prod..

[43] Snowder G.D., Glimp H.A. (1991). Influence of breed, number of suckling lambs, and stage of lactation on ewe milk production and lamb growth under range conditions. J. Anim. Sci..

[44] Morgan J.E., Fogarty N.M., Nielsen S., Gilmour A.R. (2007). The relationship of lamb growth from birth to weaning and the milk production of their primiparous crossbred dams. Aust. J. Exp. Agric..

[45] Hovey R.C., McFadden T.B., Akers R.M. (1999). Regulation of mammary gland growth and morphogenesis by the mammary fat pad: A species comparison. J. Mammary Gland Biol. Neoplasia.

[46] Hovey R.C., Aimo L. (2010). Diverse and active roles for adipocytes during mammary gland growth and function. J. Mammary Gland Biol. Neoplasia.

[47] Hoshino K., Yokoyama A.M.H., Nagasawa H. (1978). Mammary transplantation and its histogenesis in mice. Physiology of Mammary Glands.

[48] Colville T., Colville T., Bassert J.M. (2007). Chapter 18. Pregnancy, Development, and Lactation. Clinical Anatomy and Physiology for Veterinary Technicians.

[49] Anderson R.R. (1975). Mammary gland growth in sheep. J. Anim. Sci..

[50] Nørgaard J.V., Nielsen M.O., Theil P.K., Sørensen M., Safayi S., Sejrsen K. (2008). Development of mammary glands of fat sheep submitted to restricted feeding during late pregnancy. Small Rumin. Res..

